# Network Analysis of the Association between Minority Stress and Activism in LGB People from Poland

**DOI:** 10.3390/ejihpe14070122

**Published:** 2024-06-21

**Authors:** Aleksandra Krok, Zofia Kardasz, Aleksandra M. Rogowska

**Affiliations:** Institute of Psychology, University of Opole, 45-052 Opole, Poland

**Keywords:** activism, bisexual, gay, lesbian, LGB, sexual minority stress

## Abstract

(1) Background: This paper presents an analysis of the associations between minority stressors and activism in the population of lesbian, gay, and bisexual individuals in Poland. (2) Methods: The cross-sectional online-based research was conducted among 192 lesbian, gay, and bisexual (LGB) people in two groups: activists (*n* = 51) and people not engaged in activism (*n* = 141). Four scales of the Sexual Minority Stress Scale were used: Internalized Homophobia, Expectation of Rejection, Concealment, and Sexual Minority Negative Events Scale. Activism was measured using the Activism Scale and a single item asking about belonging to an activist group. (3) Results: Activists, lesbians, and gays scored significantly higher in the Sexual Minority Negative Events than people not involved in activism and bisexual individuals. Attitude toward LGBT+ activism correlated weakly and positively with Sexual Minority Negative Events and Expectation of Rejection while negatively with Internalized Homophobia. The Network Analysis showed that positive attitudes toward LGBT+ activism, the expectation of rejection, and negative events in minority stress are the most influential variables in the network model, playing a crucial role in the interaction between particular dimensions of minority stress. (4) Conclusions: Prevention and intervention programs should focus on reducing minority stress, especially negative events and rejection, especially among lesbians, gays, and people engaged in LGBT+ activism. The cooperation of politicians, lawyers, social workers, and psychologists is required to decrease homophobia and the stigmatization of people representing sexual and gender minorities.

## 1. Introduction

### 1.1. Minority Stress

Minority stress is an additional burden of stressors that affect people of socially stigmatized minorities. It results both from experienced acts of discrimination and stigmatization, as well as from the fact that people live in a majority society that imposes specific requirements on them as to norms, values, and behavior [1,2]. Research shows the negative impact of minority stress on various aspects of mental health, physical health, and close relationships among gay, lesbian, and bisexual (LGB) people [3,4,5].

Meyer’s minority stress model [6] identified four stressors related to stress processes: (1) prejudice events, (2) stigma—an expectation of rejection and discrimination, (3) concealment, and (4) internalized homophobia. These stressors are placed on a continuum, from distal (external, far from self) to proximal (close to self). The minority stress model and identified stressors have been repeatedly used in numerous studies and adapted to the specificity of minority populations [7,8,9,10,11,12,13,14,15]. 

Despite distinguishing four main stressors in Meyer’s minority stress model [6], empirical studies rarely use measurement methods that take into account all these stressors to determine the burden of minority stress. The most commonly used indicators are experiences of discrimination (stressor furthest from self) [3,16], internalized homophobia, and internalized heterosexism (stressor closest to self) [3,4,17] or strong correlates of minority stress [18]. However, little attention is paid to the intermediate processes between the experiences of discrimination and internalized homophobia, namely, the anticipation of stigma and concealment of one’s identity. The current study fills this gap in literature by examining all four dimensions of minority stress, as outlined in Meyer’s model [6].

Research shows some differences in experiencing minority stress between homosexual and bisexual individuals. Bisexual people are more likely to indicate lower social outness and higher social rejection (from parents, siblings, and friends) than homosexual people [19]. Bisexuals are also at risk for unique stressors connected with bisexuality, both in heterosexual and homosexual communities. Bisexual identity is hypothetically one of the most important factors explaining the higher risk of bisexual people experiencing mental health and substance use problems [20].

### 1.2. Understanding Activist Engagement

According to Klar and Kasser [21], activism is defined as the behavior of supporting a political cause through a variety of means. Activistic behavior ranges from more institutionalized (such as submitting a petition) to less conventional, such as civil disobedience. They define the primary goal of activism as improving society through political behavior. For many years, research focused on activism as a reaction to negative experiences and feelings of deprivation [22]. Relative deprivation, unlike absolute deprivation, comes from social comparisons. It does not evaluate if an in-group is poorly treated, but whether it is treated worse than other groups. To assess relative deprivation, an individual goes through three stages: (1) the comparison of one’s situation with another group; (2) as a result of comparisons, the feeling that a person is at a disadvantage; and (3) the assessment of observed inequality as unfair [23,24]. Relative deprivation motivates actions aimed at reducing this deprivation [24] and can be associated with intentions to engage in collective actions, both peaceful and violent [22,25,26].

Another approach using the Significance Quest Theory [22,27] focuses on the positive outcomes of activism. This theory assumes that people have a fundamental need to feel appreciated and important for their lives to be meaningful. Circumstances that lead to relative deprivation, humiliation, rejection, and unequal treatment frustrate this fundamental need. Commitment to a larger purpose increases one’s sense of personal significance by satisfying the need to belong to groups of like-minded people and demonstrating a commitment to shared values. When people experience states of humiliation or unequal treatment, they experience an aversive state of the loss of meaning, which motivates them to change the current situation. One way to regain a sense of personal meaning is to engage in social behaviors aimed at confirming the commitment to critical social values, which is the behavioral manifestation of activism. 

### 1.3. Role of Activism for Queer Individuals in the Context of Minority Stress

Activism engagement plays an essential role for queer people, especially in countries like Poland, where the legal and social situation of LGBT+ persons is far from good [28,29] and one of the worst of the European Union (EU) countries [30]. Taking into account the scale of discrimination and social resistance related to the rights of LGBT+ people in Poland, one may be tempted to say that most activism regarding LGBT rights in Poland is mainly at high risk. Activism is inextricably linked to social exposure. Although activism has been known to produce numerous positive psychological consequences, it is also commonly associated with heightened levels of stress, negative emotions, and emotional exhaustion [31,32,33,34]. Moreover, those who engage in high-risk activism may be at increased risk of verbal and physical attacks [34].

Fortunately, there are also positive effects for activism-engaged individuals. In previous years, there was some research combining minority stressors and activist engagement. Research showed that activist engagement can be considered a problem-focused way to cope with minority stress [18,35]. Findings indicate that activism allows for a change in social structures, contributing to minority stress [36,37]. Other studies focused on positive activist effects that contribute to better stress management, higher community connectedness [17], better health [38], and different aspects of well-being [21]. In addition, LGBT+ people involved in activism declared a lower degree of the internalization of heterosexism [17]. The present research will focus on the association between sexual minority stress and activist engagement among LGB people from Poland. 

### 1.4. Aim of This Study

The main goal of this study was an in-depth analysis of the relationship between activism and four minority stressors distinguished by Meyer’s [6], including internalized homophobia, the concealment of own identity, the expectation of rejection due to sexual orientation, and negative experiences due to belonging to a sexual minority. Because both activism and minority stress are culturally based, it is interesting to examine the relationship between sexual minority stress and attitude toward engagement in LGBT+ activism in a sample of sexual minorities in Poland. Sexual minority stress and attitudes toward engagement in LGBT+ activism will be compared across groups representing various sexual identification and samples of participants belonging to activist groups and those who did not engage in activism. The relationships between sexual minority stress and attitude toward engagement in LGBT+ activism will be explored using correlation and network analyses. To our knowledge, these research problems will be explored for the first time, so direct hypotheses are not assumed. This study presents an opportunity to develop strategies or methods that can be implemented to address the persistent stigma and prejudice experienced by the LGBT+ community. Furthermore, the comparison of lesbian and gay samples with bisexual participants constitutes a significant methodological advantage over previous studies.

## 2. Materials and Methods

### 2.1. Study Design and Procedure

The cross-sectional survey study was performed from 1 December 2022 to 5 October 2023 in Poland using a Google Forms online questionnaire. The invitation to participate in the study was disseminated via social media in groups associated with LGBT+ people, on the profiles of private persons from the LGBT+ community and allies, on the fan pages of places friendly to LGBT+ people, and also sent to queer groups, foundations, and non-profit organizations (NGOs) related to activities for LGBT+ people. Some respondents learned about the study from a live conversation, e.g., during various meetings related to equality activism. These people also completed the online survey.

The inclusion criteria were adults aged 18 or older who identified as bisexual or homosexual. Since minority populations experience minority stress, heterosexual identification was an exclusion criterion. Participants answered the single question “What is your psychosexual orientation?” and those who answered “heterosexual” were directed to the final section, where a message was waiting for them: “Thank you for your willingness to participate and your time! Unfortunately, you are not the target group of this study. If you still want to contribute to my research, I invite you to forward this form to non-heterosexual people”. Their cisgender/transgender identity did not distinguish participants, bearing in mind that an adequate study of minority stressors experienced by transgender people would require other theoretical models, taking into account their unique experiences [39].

An a priori power analysis was conducted using G*Power ver. 3.1.9.6 [40] to calculate the minimum number of participants needed to test the group differences (Student’s *t*-test) and associations between variables (correlation *r*). Results indicated that the required sample size to achieve 80% power for detecting a medium effect (Cohen’s *d* = 0.50) for Student’s *t*-test, at a significance criterion of α = 0.05, was *n* = 64 for each of the two groups (the minimum total sample is *N* = 128). To determine the medium correlation effect (*r* = 0.30), at a significance criterion of α = 0.05 and 80% power, we needed a total sample of 67 people. Initially, 233 people responded to the invitation, but 41 were excluded because of their other-than-LGB sexual identification (*n* = 12), young age (*n* = 21), and missing data (*n* = 8, more than 5% of missing data in the survey). The final sample included 192 lesbian, gay, and bisexual adults. The post hoc analysis, which takes into account the current unequal sample sizes between subgroups, showed that the power in Student’s *t*-test was 93% for differences between bisexual, lesbian, and gay participants, 86% between activists and non-activists, and 99% for correlation analysis. Thus, the obtained sample size of *N* = 192 is more than adequate.

### 2.2. Participants 

A sample of 192 adults aged between 18 and 44 (*M* = 25.34, *SD* = 6.15) participated in the study, including 94 bisexual individuals and 98 lesbians and gays. Most were women with secondary education and living in a city of 100,000 to 500,000 inhabitants (Table 1).

### 2.3. Measures

The questionnaire Google Forms was designed to measure minority stress and activism. The first part of the questionnaire was information about the study and informed consent. Next, demographic information and four scales of minority stress were presented, including Internalized Homophobia (IH), Expectation of Rejection (ExR), Concealment (Clm), and Sexual Minority Negative Events (SMNE). All minority stress scales are part of the Sexual Minorities Stress Scale (SMSS), developed by Peter Goldblum, James Dilley, and Matthew Skinta based on Meyer’s model of sexual minority stress [1] and with Ilan Meyer’s contribution. The SMSS was developed as a clinical instrument to identify LGB clients with clinically significant sexual minority stress for the research project of the Center for LGBTQ Evidence-Based Applied Research (CLEAR) in collaboration with the UCSF AIDS Health Project in San Francisco [41]. Polish validation and adaptation were performed by Iniewicz et al. [12]. Finally, participants completed the Activism Scale [42,43] as the last part of the survey.

#### 2.3.1. Demographic Survey

Demographic questions were related to the age of the participants (the number of years, continuous variable), sexual identification (Bisexual, Lesbian, Gay, Other), gender identification (Women, Men, Nonbinary), education (Primary, Vocational, Secondary, Higher), and place of residence (Village, City up to 20,000 inhabitants, City of 50,000 to 100,000 inhabitants, City of 100,000 to 500,000 inhabitants, City from 500,000 up to one million inhabitants, City with over 1 million inhabitants). 

#### 2.3.2. Internalized Homophobia

Internalized Homophobia (IH) is assessed on a 10-item scale. Goldblum et al. [41] developed the IH scale from similar scales [44,45] and Iniewicz et al. [12] validated the Polish. The items refer to the discomfort of same-sex attraction and the extent to which a gay or bisexual person rejects their sexual orientation and attempts to avoid emotional attraction or sexual desire (e.g., “Have you tried to stop being attracted to persons of the same sex?”). Participants rate, on a 4-point Likert scale of responses, how often a given behavior was presented (from 1 = “Never” to 4 = “Often”). Higher scores mean a higher level of internalized homophobia. The internal reliability (Cronbach’s α) was 0.84 in the Polish validation study [12] and 0.82 in the current sample.

#### 2.3.3. Expectations of Rejection

The Expectation of Rejection scale (ExR) was developed [41] on the basis of previous scales [44,46] and validated in Polish by Iniewicz et al. [12]. The ExR consists of six items, measuring the extent to which a homosexual or bisexual person experiences resentment from others and expects rejection and stigmatization (e.g., “Most people believe that a person like you cannot be trusted”). The response options range from 1 to 4 (where 1 = “Strongly disagree” and 4 = “Strongly agree”), and higher scores indicate higher expectations of rejection. The reliability (Cronbach’s α) of the ExR was 0.86 in the previous study [12] and 0.88 in the present research.

#### 2.3.4. Concealment

The Concealment (Clm) scale was developed [41,47] and validated in Polish [12] as a six-item measure of the extent to which individuals deliberately conceal their sexual orientation from others (e.g., “I am concealing my sexual orientation by avoiding contact with other LGB individuals”). Participants rate, on a 5-point Likert scale (from 1 = “Not at all” to 5 = “All the time”), how often the given behavior was presented, and a higher score indicates a higher level of concealment. The internal consistency of the Clm scale was Cronbach’s α = 0.83 in the Polish validation [12] and 0.76 in the current sample.

#### 2.3.5. Sexual Minority Negative Events

The Sexual Minority Negative Events (SMNEs) is a 26-item scale to assess sexual orientation-related stressors experienced by homosexual and bisexual people [12,41]. The SMNE includes stressors experienced by self (e.g., “I was treated unfairly by peers or siblings”) or other people (e.g., “I heard negative statements about LGB or gender nonconforming people”) and events related to the risk of sexually transmitted disease (e.g., “I have been diagnosed with HIV or other chronic sexually transmitted diseases, such as hepatitis C”). For each of the first 22 items, the scale contains four possible answers: “Before turning 18 at home” = 1; “Before turning 18 in school” = 1; “After turning 18 or later in life” = 1; and “Did not happen to me” = 0. For items 23–26, the answers are “Yes” = 1 and “No” = 0. The score for each item is the sum of items, which means the answer range is 0–3 for items 1–22 and 0–1 for items 23–26. Higher scores indicate more adverse events experienced. The Cronbach’s α reliability coefficient was 0.84 in the previous [12] and 0.87 in the present study.

#### 2.3.6. Activism

The Activism Scale contains three items, developed to examine the attitude to activities for LGBT+ people [42,43]. A respondent answers, on a seven-item Likert scale (1 = “I strongly disagree”, 7 = “I strongly agree”), the following statements: (1) “I want to get involved in activities to strengthen the interests of LGBT+ people in Poland”; (2) “I do not see the need to participate in activities aimed at strengthening the position of LGBT+ people in Polish society” (scoring reversed); and (3) “I will take part in a collective action for Polish LGBT+ people”. A higher score means a more positive attitude toward engagement in LGBT+ activism. The reliability of the AS was Cronbach’s α = 0.84. 

Also, we used a single question, “Do you belong to any activist group?” with two response options (0 = “No” and 1 = “Yes”). This item was adapted from previous research [17] to distinguish a group of activists from non-activists.

### 2.4. Statistical Analysis

Initially, parametric properties of continuous variables (IH, ExR, Clm, SMNE, and AS scales) were examined using mean (*M*), standard deviation (*SD*), median (*Mdn.*), skewness, and kurtosis. Since skewness and kurtosis ranged between ±1.50, and the sample size was quite large (*N* = 192), we decided to use parametric tests to verify hypotheses. Therefore, independent-samples Student’s *t*-test was performed to examine differences between groups of people who differed in sexual identification (bisexual, homosexual), and activism (activists, non-activists) in minority stress (IH, ExR, Clm, SMNE) and activism scales (AS). Pearson’s correlations were used to find associations between all continuous variables (IH, ExR, Clm, SMNE, and AS scales). Finally, network analysis (NA) with the extended Bayesian information criteria and graphical-least-absolute-linkage-and-selection operator (EBICglasso) as an estimator was performed to examine a specific configuration of relationships between minority stress scales and activistic attitude. NA focuses on understanding and analyzing the relationships and interactions between individuals, groups, and variables in a social context. All statistical analyses were performed using JASP ver. 0.16.1.0. software for Windows.

## 3. Results

### 3.1. Differences between Homosexual and Bisexual People in Minority Stress and Activism

The independent-samples Student’s *t*-test showed that lesbian and gay participants experience significantly more stressful events related to their sexual identification (assessed on the SMNE scale, *p* < 0.01) than bisexual individuals (Table 2). However, the effect size for these differences was small (Cohen’s *d* = 0.42). No significant differences were found for the other variables, including internalized homophobia, the expectation of rejection, concealment, and activistic attitude. 

### 3.2. Differences between Activists and Non-Activists in Minority Stress and Activistic Attitude

Student’s *t*-test examined differences between people engaged in activism and their non-activist peers. Activists scored significantly higher on the Activist Scale (*p* < 0.001, large effect size) and experienced adverse stressful events related to belonging to a sexual minority community (SMNE; *p* < 0.001, medium effect size) more than people not involved in activism (Table 3).

### 3.3. Correlations between Minority Stress and Activism

Pearson’s correlation examined associations between minority stress scales and activism (Table 4). Activism is positively related, but at a low level, to adverse stressful events and expectations of rejection, and negatively to internalized homophobia. A low positive correlation was presented between internalized homophobia and expectations of rejection and concealment. In addition, expectations of rejection were related positively to concealment and sexual minority negative events. 

### 3.4. Network Analysis for Associations between Minority Stress and Activism

**Figure 1:**
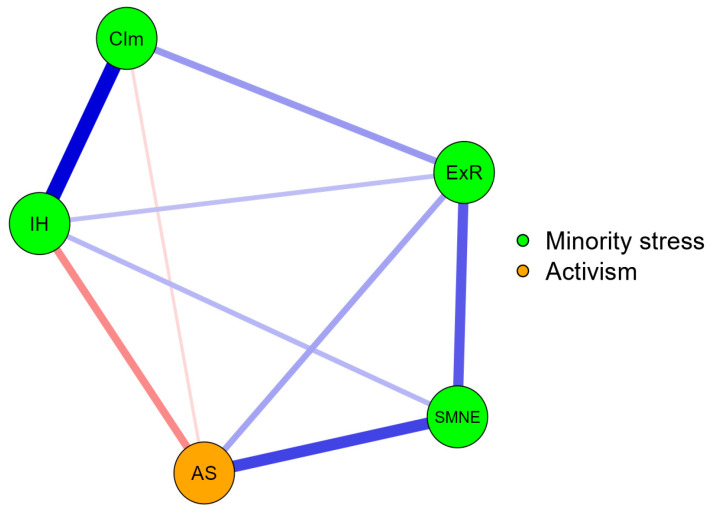
Network analysis for associations between minority stress and activism. IH = Internalized Homophobia, ExR = Expectations of Rejection, Clm = Concealment, SMNE = Sexual Minority Negative Events, AS = Activism Scale.

**Figure 2:**
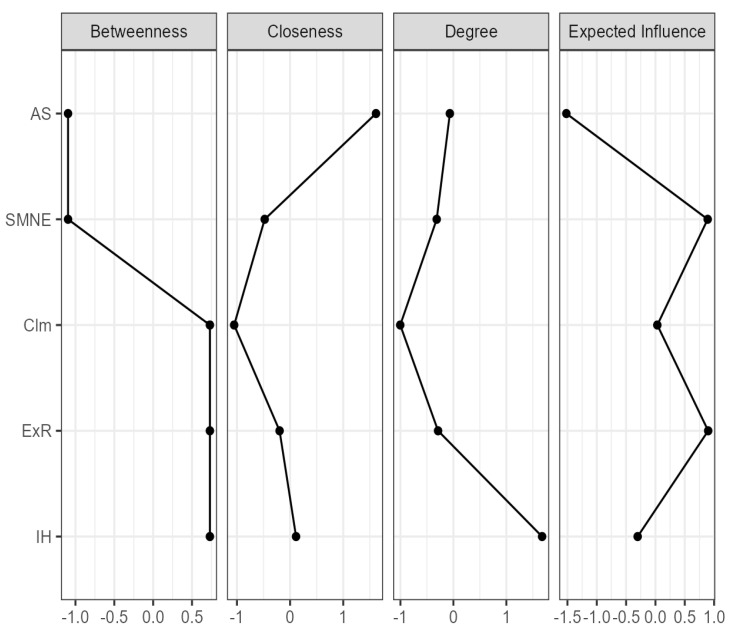
Centrality plot for associations between minority stress and activism. IH = Internalized Homophobia, ExR = Expectations of Rejection, Clm = Concealment, SMNE = Sexual Minority Negative Events, AS = Activism Scale.

Network Analysis assumes that structures of relationships can be represented as networks of interconnected nodes and ties (edges or connections) between these nodes. Colors indicate the association’s direction; blue represents a positive association, while red indicates negative relationships. The weighted network between nodes is represented by magnitude. The thicker the lines between the nodes, the stronger the relationship. The closeness between nodes also shows the strength of correlations. Moreover, we used several centrality indices (i.e., betweenness, closeness, degree, and the expected influence) to identify the most critical variables and their role in the network and assess the variables’ relevance in a model (Figure 1 and Figure 2).

The network structure of associations between minority stress scales and activism is visualized in Figure 1. NA showed that activism is strongly, closely, and positively related to minority stress negative events, and positively but weakly related to the expectation of rejection. Minority stress negative events are a bridge between activism and the expectation of rejection. In contrast, a negative and medium association is presented between activism and internalized homophobia, while a much weaker and negative association is demonstrated between activism and concealment. Internalized homophobia plays a bridge role between activism and concealment. Among minority stress scales, the chain of interconnections leads from minority stress negative events, through the expectation of rejection and concealment, to internalized homophobia. 

The study also used the centrality indices to find the crucial variables for the network model (Figure 2). Betweenness represents the degree of connectivity, considered the number of times a node is part of all the pairs of the associated nodes in the network. In NA, betweenness centrality is a measure that quantifies the importance of a node in a network based on its position as a bridge or intermediary between other nodes. It helps identify nodes critical in facilitating communication, information flow, or influence between network parts. Three scales of minority stress, IH, ExR, and Clm, revealed the most important positive variables regarding the number of connections within the network model. These scales act as bridges, connecting all minority stress scales. Also, SMNE and AS presented high negative betweenness values, suggesting they may have the most influence or control over the interaction processes in the network. Due to their strategic position, activism, and minority stress, negative events can be pivotal in mediating relationships between other nodes. 

Closeness centrality is a measure that quantifies how close a node is to all other nodes in the network, considering the shortest paths between them. It identifies more central nodes regarding accessibility, indicating their potential to spread information or influence efficiently. In the NA (Figure 2), activism showed the highest closeness centrality value, which indicates that AS has shorter average distances to all other nodes in the network. Also, SMNE and Clm are more centrally located, can access information or communicate with other nodes more quickly, and can efficiently spread information or messages across the network. The three nodes, AS, Clm, and SMNE, can serve as central hubs for communication.

Degree centrality (or strength) is the sum of all the paths that connect the nodes in terms of the mean of correlation weights. In NA, the degree is a fundamental measure used to interpret the importance and connectivity of nodes in a network. The highest strength of centrality in the NA (Figure 2) demonstrates internalized homophobia (positive influence in the network) and concealment (negative influence in the network). These variables are highly connected with other nodes and play a significant role in the network. 

Finally, the expected influence shows the essential variables that can act as a bridge between the adjacent nodes. For minority stress scales (Figure 2), the most influential are minority stress adverse events and the expectation of rejection. On the other hand, a negative influence was found in activism. In contrast, considering the expected influence measure, concealment and internalized homophobia do not play a bridge role in the present network.

## 4. Discussion

The present study is the first attempt to examine the relationship between minority stress and activist involvement among the lesbian, gay, and bisexual population from Poland. The results of this study are particularly important due to the specific socio-political context regarding the situation of queer people in Poland. The findings contribute to the growing body of research in the field of mental health, social situation, and the empowerment of LGBT+ persons around the world, showing results specific to the Polish culture. 

### 4.1. Intergroup Differences

A comparison of LGB people belonging to activist groups and LGB people not belonging to activist groups revealed two significant differences. Firstly, activists (defined as people who declared belonging to at least one activist group) showed significantly higher attitudes toward engagement in collective actions aimed at improving the situation of LGBT+ people in Poland. This result may be consistent with research indicating that activist identity may be a significant predictor of intentions to engage in activism, both high- and low-risk [36,48].

Secondly, the group of activists exhibited a significantly higher level of SMNE than the group of people not belonging to activists. There are two possible interpretations of this relationship. First is that people with higher scores in negative events due to belonging to the LGBT+ community may be more willing to engage in activities aimed at changing the situation, either by reducing the feeling of relative deprivation [22,24,25,26] or the need to regain personal meaning according to the Quest for Significance theory [22,27]. A second explanation is that activistic LGB people in Poland are highly exposed to experiencing negative events and discrimination due to their minority identity. Participation in activism is an additional opportunity to experience the adverse effects of belonging to the sexual minority, like heightened minority stress, stigmatization, and rejection. However, it cannot be clearly stated whether it is the experience of negative events that leads to involvement in activism, or whether activist involvement contributes to the experience of negative events related to belonging to a sexual minority. This issue is worth exploring in future research. 

Previous studies comparing differences in minority stress among LGB people indicated a higher burden in bisexual people [19,20]. However, the data in the present study did not confirm a similar tendency. The differences between bisexual participants and lesbian and gay were insignificant, except for the score on the SMNE, indicating a higher level of this type of stressor in the group of homosexual people, contrary to the predictions. This inconsistency may be related to cross-cultural differences. It is plausible that bisexual individuals in Poland can effectively conceal their sexual identity, leading to a dual existence as a heterosexual person in conservative social settings and as a bisexual person in the LGBT+ circle. Therefore, bisexuals may experience less adverse consequences of belonging to the minority population than people who have clearly revealed themselves as gay or lesbian. It is also possible that the group of LGB people studied here is not representative of the minority population due to measurement errors related to the small sample size and the online survey form addressed to selected Facebook groups. However, future research is required to verify these speculations.

### 4.2. Associations between Sexual Minority Stress and Activism

The study results showed weak positive correlations of SMNE with the expectation of rejection and attitude toward engagement in LGBT+ activism. Negative experiences of discrimination related to one’s sexual identity and the expectation of rejection due to this identity, in accordance with the theories presented earlier, may hypothetically motivate people to act for social change. It seems essential that this relationship occurred for the entire sample (not only for activists, but also for non-activists). There was also a weak negative correlation between internalized homophobia and intentions of activist involvement. This result corresponds to previous research that indicated a lower degree of internalization of heterosexism among people involved in activism [17]. 

For the first time, NA was used to assess specific interactions between particular dimensions of sexual minority stress and attitude toward LGBT+ activistic activity. The chain of interconnections leads from minority stress negative events, through an expectation of rejection and concealment, to internalized homophobia. This pattern partially reflects the continuum of distal-to-proximal stressors originally proposed by Meyer [1], with one significant difference regarding the exchange of internalized homophobia and concealing one’s sexual orientation. However, the most important result of NE is the structure of interconnections between activistic attitudes and particular dimensions of sexual minority stress. Those LGB people who scored high in a positive attitude toward activism to LGBT+ society frequently experience negative events related to sexual minorities and expectations of rejection. At the same time, simultaneously, they perceived low internalized homophobia and concealment. This result may suggest that the primary motivation for belonging to an activist organization is to rebel against homophobia and stigmatization, and to improve the situation of people from sexual minorities in Poland. 

This assumption seems to confirm the analysis of centrality measures. Taking into account betweenness, internalized homophobia, expectations of rejection, and concealment presented the most connections in the NA, acting as bridges between variables. On the other hand, SMNE and activism were the most influential in the interaction processes in the network. Furthermore, the closeness centrality measure showed that activism, concealment, and SMNE play a central role in communication in the network. In addition, internalized homophobia presented the strongest positive influence in the network, while concealment had the strongest negative influence in the network, showing their high importance in the set of variables. These variables have a large number of immediate neighbors, making them potentially influential in spreading information, coordinating interactions, or transmitting resources. Finally, minority stress adverse events and expectation of rejection revealed the highest expected influence in the NA. These variables are essential and act as a bridge between all other dimensions of minority stress and activism.

Interestingly, activism showed a negative expected influence in the model of interconnections. This result suggests that activism can be considered an effective way to cope with sexual minority stress. In contrast, minority stress adverse events and expectations of rejection are crucial for increasing sexual minority stress. We could hypothesize that activist involvement can help transform experiences of oppression, so that they will not lead to the internalization of negative beliefs about one’s sexual orientation and its concealment. Unfortunately, although this interpretation is optimistic, we cannot accept it without considering the alternative scenario. Assuming the interpretation that involvement in activism leads to an increased burden of SMNE, even greater than in the rest of the queer population, indicates the need for specific support for queer activists to help them cope with potentially traumatic discrimination events and prevent burnout—rather than encouraging LGBTQ people to do activism as a way of dealing with systemic oppression.

### 4.3. Limitation of the Study

Although results seem promising for future research, some issues prevent generalization. This research is unable to extend its findings to the broader LGB population due to limitations in the sample size and non-random sampling procedures. The sample size was not large, so it could not be representative of the LGB population in Poland. Also, online studies exclude those who do not use the Internet, which is the following source limitation of this study. In addition, the LGB people participated in the study, so it is unclear whether the same results could be presented among people representing other sexual (e.g., pansexual, intersexual, asexual) and gender identities (e.g., transgender, agender, fluid gender, queer, nonbinary). More research is required, especially cross-cultural studies in the broad international society of LGBT+ people, to verify the present study results.

Furthermore, the study used self-report questionnaires to measure minority stress and attitudes toward engagement in LGBT+ activism. Future research should explore these variables using qualitative research methodologies, with the aim of verifying the current findings through alternative research designs. Moreover, longitudinal studies could potentially elucidate the causal or reciprocal associations between the dimensions of minority stress and attitudes toward engaging in LGBT+ activism. Further research should examine gender differences in minority stress and activism. Research needs to be conducted to explain why LGBT+ people involved in activism experience more negative events compared to people who are not activists. The present investigation failed to consider pertinent factors such as religiosity, trust in governmental bodies and authorities, political leanings, and the sense of security within the nation. Subsequent research ought to account for these variables.

## 5. Conclusions

A novel finding suggests that LGB individuals engaged in LGBT+ activism, as well as lesbian and gay people in Poland, can experience higher levels of sexual minority negative events compared to those who are inactive and bisexuals, respectively. These target groups should receive support, as well as intervention and therapeutic programs focused on decreasing minority stress. As shown by the NA, involvement in activism toward the LGBT+ society may be a practical tool or a specific coping strategy that mediates between negative events related to minority stress and the internalization of homophobia, expectation of rejection, and concealment of identity. However, engagement in activism in the LGBT+ society can also increase the number of negative events related to this activity. Therefore, people engaged in LGBT+ activism should be particularly protected by the majority of society. 

The structure of NA in the present study confirmed the minority stress model by Meyer [1], indicating that LGB people in Poland experience a continuum of distal-to-proximal stressors related to their minority status. It is important to note that minority stress and activism are very delicate and complex issues, combining aspects of social psychology, clinical psychology, politics, law, and other fields. Conclusions from this research may be different for social movement researchers, clinicians supporting LGBTQ+ people, queer communities, and others supporting queer activists. A further study could explore the link between the experience of oppression and intentions to engage in social change, as well as point to the importance of membership in activist groups in predicting attitudes and intentions of future activism involvement.

Perhaps the key to understanding the two conclusions may be to view these findings not as mutually exclusive opposites, but as complementary elements. Undoubtedly, it is crucial to acknowledge the beneficial outcomes of activism, while simultaneously grappling with the potential for traumatic ramifications for individuals involved in these efforts. Hence, it is essential to extend interventions beyond bolstering the resilience of LGBTQ individuals, particularly those engaged in activism, at the individual level, and instead address the communal and systemic dimensions as well.

The NA showed that positive attitudes toward LGBT+ activism and minority stress negative events were the most crucial for the interaction between all variables within the NA model. In addition, minority stress negative events and the expectation of rejection revealed the highest expected influence in the NA. Therefore, prevention and intervention programs should be aimed at reducing the number of negative events and rejection of LGBT+ people by the Polish society. Politicians, lawyers, social workers, and psychologists should cooperate on the development of a coherent program to counteract homophobia and the stigmatization of people from sexual and gender minorities.

## Figures and Tables

**Table 1:** Participant characteristics.

Variable	Categories	*n*	%
Sexual Identification	Bisexual	94	48.96
	Lesbian and gay	98	51.04
Gender Identification	Women	111	57.81
	Men	70	36.46
	Nonbinary	11	5.73
Education	Primary	12	6.25
	Vocational	3	1.56
	Secondary	96	50.00
	Higher	81	42.19
Place of residence	Village	24	12.50
	City up to 20,000 inhabitants	8	4.17
	City 20 to 50 thousand inhabitants	19	9.90
	A city of 50,000 to 100,000 inhabitants	14	7.29
	A city of 100,000 to 500,000 inhabitants	49	25.52
	City from 500,000 up to one million inhabitants	39	20.31
	A city with over 1 million inhabitants	39	20.31

**Table 2:** Independent-samples Student’s *t*-test between people of homosexual and bisexual identification on scales of minority stress and activism.

Variable	Lesbian & Gay (*n* = 98)		Bisexual (*n* = 94)		*t* (190)	*p*	*d*
	*M*	*SD*	*M*	*SD*			
Internalized Homophobia	16.10	5.37	16.61	5.00	–0.67	0.502	–0.10
Expectation of Rejection	12.90	3.95	12.18	4.21	1.22	0.225	–0.10
Concealment	11.97	4.80	11.80	4.40	0.26	0.797	0.04
SM Negative Events	15.67	10.29	11.63	6.97	3.18	0.002	0.46
Activism Scale	14.91	5.81	15.30	5.24	–0.49	0.626	–0.07

Note. SM = Sexual Minority.

**Table 3:** Independent-samples Student’s *t*-test between activists and non-activists on scales of minority stress and activism.

Variable	Non-Activist (*n* = 141)		Activist (*n* = 51)		*t* (190)	*p*	*d*
	*M*	*SD*	*M*	*SD*			
Internalized Homophobia	16.31	5.13	16.45	5.38	–0.16	0.870	–0.03
Expectation of Rejection	12.32	3.94	13.18	4.44	–1.29	0.200	–0.21
Concealment	11.64	4.46	12.57	4.95	–1.24	0.216	–0.20
SM Negative Events	12.24	8.47	17.71	9.38	–3.84	<0.001	–0.63
Activism Scale	13.76	5.49	18.80	3.64	–6.10	<0.001	–1.00

Note. SM = Sexual Minority.

**Table 4:** Pearson’s correlations between scales of minority stress and activism.

Scale	Range	*M*	*SD*	IH	ExR	Clm	SMNE
1. IH	9–36	16.35	5.18				
2. ExR	6–24	12.55	4.08	0.20 **			
3. Clm	6–27	11.89	4.60	0.45 ***	0.23 **		
4. SMNE	0–47	13.69	9.03	0.13	0.35 ***	0.08	
5. AS	3–21	15.10	5.52	−0.17 *	0.21 **	−0.10	0.33 ***

Note. IH = Internalized Homophobia, ExR = Expectations of Rejection, Clm = Concealment, SMNE = Sexual Minority Negative Events, AS = Activism Scale. * *p* < 0.05, ** *p* < 0.01, *** *p* < 0.001.

## Data Availability

The data reported in this research are available on request from the corresponding author.
